# Cost-effectiveness of Mental Health Interventions in Schools

**DOI:** 10.1192/j.eurpsy.2025.127

**Published:** 2025-08-26

**Authors:** D. McDaid, A.-L. Park

**Affiliations:** Care Policy and Evaluation Centre, Department of Health Policy, London School of Economics and Political Science, London, United Kingdom

## Abstract

**Abstract:**

Children and young people spend much time in school and therefore the school setting provides an important setting in which to intervene to promote, protect and enhance mental health. This presentation will provide an overview of the importance of making an economic case for action within schools. A scoping review has been undertaken to map the types of interventions that have been subject to cost-effectiveness evaluation. Aspects of the strengths and weakness of the existing evidence are presented. Implementation and sustainability challenges are also discussed, given the need for collaboration between the health and education sectors.

**Disclosure of Interest:**

None Declared

